# Evaluating professionals’ adaptations before and after a decision support intervention “the Adaptation and Fidelity Tool” (A-FiT)—A longitudinal within-person intervention design

**DOI:** 10.1177/26334895251334552

**Published:** 2025-04-13

**Authors:** Johanna Zetterlund, Henna Hasson, Ulrica von Thiele Schwarz, Margit Neher, Emmie Wahlström

**Affiliations:** 1 Department of Health, Care and Social Welfare, 8177 Mälardalen University, Västerås, Sweden; 2 Department of Learning, Informatics, Management and Ethics, 27106 Karolinska Institutet, Stockholm, Sweden; 3 Centre for Epidemiology and Community Medicine, Region Stockholm, Stockholm, Sweden; 4 School of Health and Welfare, 3694 Halmstad University, Halmstad, Sweden

**Keywords:** adaptation, treatment adherence, evaluation, evidence-based, use of evidence, sustainment, implementation supports, implementation, decision making, treatment fidelity, practice context

## Abstract

**Background:**

Implementing evidence-based interventions (EBIs) in practice requires balancing fidelity and adaptation to suit new contexts. Careful considerations are needed to maintain the core elements for effectiveness while ensuring fit with new contexts. The Adaptation and Fidelity Tool (A-FiT) intervention addresses this challenge by providing support for professionals using EBIs in the sustainment phase of implementation. This study evaluates the A-FiT intervention and examines how professionals delivering an EBI manage fidelity and adaptation during the sustainment phase of implementation, before and after the intervention. Method Short, structured interviews were repeatedly conducted with 14 professionals delivering an EBI (*n* = 127). Data was analyzed using deductive content analysis focusing on adaptation types, planning, intentionality, and fidelity consistency. The adaptations were counted and compared before versus after the A-FiT intervention using a chi^2^-test.

**Results:**

The professionals made about the same number of adaptations before and after the A-FiT intervention. However, after the intervention, significant changes in the type and intentionality of the adaptations were observed. Changes in type consisted of fewer “removing,” “substituting,” and “integrating another framework” adaptations and more “loosening structure” and “departing from the intervention” adaptations. Regarding intentionality, fewer planned adaptations with the intention of improving the EBI effects were made, while adaptations made for practical reasons, both planned and unplanned, increased after the A-FiT intervention. No statistical change was found regarding fidelity consistency.

**Conclusions:**

The findings indicate increased awareness about fidelity and adaptation among the group leaders, resulting in fewer planned adaptations to enhance program effects and more practical adaptations to address context challenges. The A-FiT intervention appears to help professionals in their management of fidelity and adaptations when delivering EBIs. The study underscores the importance of understanding adaptations in their context, purpose, and impact (intended and unintended) on the outcome/value.

## Plain Language Summary

***Adaptations Professionals Make of an Evidence-Based Intervention and the Evaluation of the Adaptation and Fidelity Tool Aiming to Support Professionals in Balancing Fidelity and Adaptation.*** This study explored what kind of adaptations group-leaders of a parental education program make when delivering the program to parents, before and after receiving the Adaptations and Fidelity Tool (A-FiT) intervention. The A-FiT intervention was developed to support professionals in fidelity and adaptation management when working with evidence-based interventions in practice. This support is important since professionals are expected to achieve a balance, performing the challenging task of trying to adhere to the intervention manual to make the intervention effective, but at the same time adapting the intervention to make it fit the context the professionals are working in, without guidance on how to do so. The study found that the A-FiT intervention seemed to increase awareness about adaptations, leading to fewer planned adaptations aimed at enhancing program effects and more practical adaptations to address context challenges. The study emphasized recognizing the value of adaptations for reaching diverse populations.

## Background

There is a balancing act between fidelity (the degree to which an intervention was implemented as prescribed or intended; Proctor et al., 2023) and adaptation (any addition, subtraction, or modification made to the original method; Moore et al., 2013), when maintaining EBIs in practice (Rabin et al., 2018). This maintenance occurs during the sustainment phase of implementation, where EBIs often are adapted by professionals delivering the EBI to the end users to ensure a continuous fit between the EBI and different contexts (Aarons et al., 2012; Bäck et al., 2020; Mosson et al., 2017; von Thiele et al., 2019). Still, the effects of adaptations on outcomes are inconclusive (Olsson et al., 2023), and high fidelity is recommended to ensure the effectiveness of the EBI (Durlak & DuPre, 2008). Therefore, careful consideration is needed when managing adaptations and fidelity to remain true to the EBI's core elements—its forms and functions (Perez Jolles et al., 2019) that make the EBI effective (Gearing et al., 2011)—while ensuring fit with new contexts. In earlier implementation phases, such considerations about fidelity and adaptations are often made in consultation with researchers and/or program specialists. However, during the sustainment phase, the professionals delivering the EBIs are solely responsible for these considerations. As these considerations require a complex balancing act, professionals struggle with not being able to follow their beliefs about how to deliver an intervention, but also experience uncertainties regarding the consequences of made adaptations, as well as fear of drifting from the EBI (Zetterlund et al., 2022).

The complexity of and professionals’ struggle with adaptations and fidelity show that professionals might need additional support when maintaining EBIs in practice. To facilitate the maintenance of EBIs and generate better outcomes there is a need to understand and develop knowledge about the sustainment phase further (Fleiszer et al., 2015; Glasgow & Chambers, 2012; Hailemariam et al., 2019; Shelton et al., 2018). A central aspect of this need is to understand why, when, and how professionals make different types of adaptations, as such knowledge might guide how to support professionals in balancing fidelity and adaptations (Aarons et al., 2012; Moore et al., 2013; Stirman et al., 2013).

Studies show that professionals are prone to making spontaneous adaptations due to sudden contextual constraints, for example, time pressure (Aschbrenner et al., 2020; Kakeeto et al., 2017; Moore et al., 2013; Stirman et al., 2013). It is also common for professionals to make adaptations without awareness of how the adaptations will affect the EBI's core elements (Wiltsey Stirman et al., 2015), or client outcomes (Cooper et al., 2016) and without planning (Stirman et al., 2015), that is, they are made without reflection on consequences or are unintentional. Making these types of unreflective adaptations can increase the risk of the EBI becoming ineffective, unsafe (Chambers et al., 2013; Kumpfer et al., 2018), unequal, or harmful (Elliott & Mihalic, 2004; Kumpfer et al., 2018). Such adaptations contrast with the recommended approach in how to handle adaptations, which emphasizes planned, fidelity-consistent adaptations with a clear purpose, aligned with the intervention's goals (Moore et al., 2013) and designed to reach the desired values of the EBI (von Thiele Schwarz et al., 2019).

The gap between how adaptations should be handled and how professionals often make them, along with the finding of their struggle with adaptations and fidelity, highlights the need for tools to help professionals make intentional, fidelity-consistent adaptations (Hasson & von Thiele Schwarz, 2017). This support is especially important in the sustainment phase as support available in the earlier phases of implementation has often been phased out (Escoffery et al., 2019), leaving professionals using EBIs facing fidelity and adaptation issues without support. To address this need, the A-FiT intervention was developed (Hasson et al., 2020, 2023; von Thiele Schwarz et al., 2021). A-FiT aims to support professionals in managing fidelity and adaptation when delivering an EBI and in making planned, intentional adaptations in line with the EBÍs core elements, based on the value the professionals want to achieve. A-FiT is grounded in the Planned Adaptation Model (Lee et al., 2008), the Useful Evidence Model (Hasson & von Thiele Schwarz, 2017) and is guided by the Value Equation Framework (von Thiele Schwarz et al., 2019). Recently, A-FiT was tested within social services in Sweden during the early phase of EBI implementation with promising results. The professionals who received A-FiT perceived it as relevant for managing fidelity and adaptations and described improvements in their knowledge concerning fidelity and adaptations (Hasson et al., 2023). However, how A-FiT can support professionals during the sustainment phase has not been investigated, nor whether A-FiT could help professionals in the actual managing of fidelity and adaptations, that is, if the professionals could be supported in making planned, intentional, fidelity-consistent adaptations when using EBIs.

## Aim and Research Questions

The study aims to describe and compare the adaptations that professionals make of an EBI before and after receiving the A-FiT.

The following research questions were addressed:

1. How many and what kind of adaptations do professionals make in terms of type, planning, intentionality, and fidelity-consistency?
2. How does A-FiT influence professionals’ adaptations of the EBI?

## Method

### Design

The study has a longitudinal within-person intervention design, using a single-phase mixed method approach. It focuses on professionals’ management of fidelity and adaptations by studying and comparing what kind of adaptations group-leaders of an evidence-based parental program called All Children in Focus (ABC; Ulfsdotter et al., 2014) make in the sustainment phase before and after they received A-FiT. The study is part of a larger project studying fidelity and adaptations from the professionals’ perspective (von Thiele Schwarz et al., 2021). The reporting of this study was guided by the Template for Intervention Description and Replication checklist (Hoffman et al., 2014), and the Transparent Reporting of Evaluation within Nonrandomized Designs statement (des Jarlais et al., 2004).

### Case-Setting

ABC is a widely adopted universal health promotion parenting program in Sweden, for example, in school settings and social services. It teaches parents strategies in how to handle challenging situations in their parenthood with the goal of improving the parent–child relationship, the parental everyday experience, and promoting children's development. The manual-based program is delivered by trained group-leaders (e.g., teachers, social workers, etc.) to groups of up to 14 parents of children aged 3–12 years. ABC includes five sessions; four weekly or biweekly sessions and one booster session held 2–3 months after the last weekly session. Each 2.5 hr session includes lectures, discussions, role plays, movies, parent manuals, and homework. Based on Behavior Analysis and Social Learning Theory, ABC is cost-effective (Ulfsdotter, 2016), and efficacious in improving children's and parents’ health (Ulfsdotter et al., 2014). For further details on ABC, see Lindberg et al. (2013).

### The A-FiT Intervention

A-FiT is a two-part digital workshop, held 1–2 weeks apart, designed to support professionals in managing fidelity and adaptation when delivering an EBI and in making planned, intentional adaptations in line with the EBI's core elements. The workshops, 3 hr each include short lectures about implementation, fidelity, and adaptation in combination with practical teamwork and individual work using structural worksheets. The development of A-FiT is presented in detail elsewhere (Hasson et al., 2023).

To ensure fit of A-FiT with the current study some changes were made to the original design of A-FiT. Additional information about ABCs context, implementation strategies, and intended outcomes of ABC were added to the A-FiT worksheets. The workshops (12 in total) were delivered to four to eight group-leaders at a time, between August 2021 and May 2022 in Sweden, using a video conference platform, and was guided by two workshop leaders (UvTS and JZ). These leaders, with backgrounds in psychology and public health, possessed facilitation skills and expertise in implementing EBIs. They were well-versed in the implementation frameworks and models (Hasson & von Thiele Schwarz, 2017; Lee et al., 2008; von Thiele Schwarz et al., 2019) used in A-FiT and had led similar workshops previously. Pilot testing of A-FiT was conducted with four ABC group-leaders and three ABC-educators during the spring of 2020.

### Participants and recruitment

**Figure 1:**
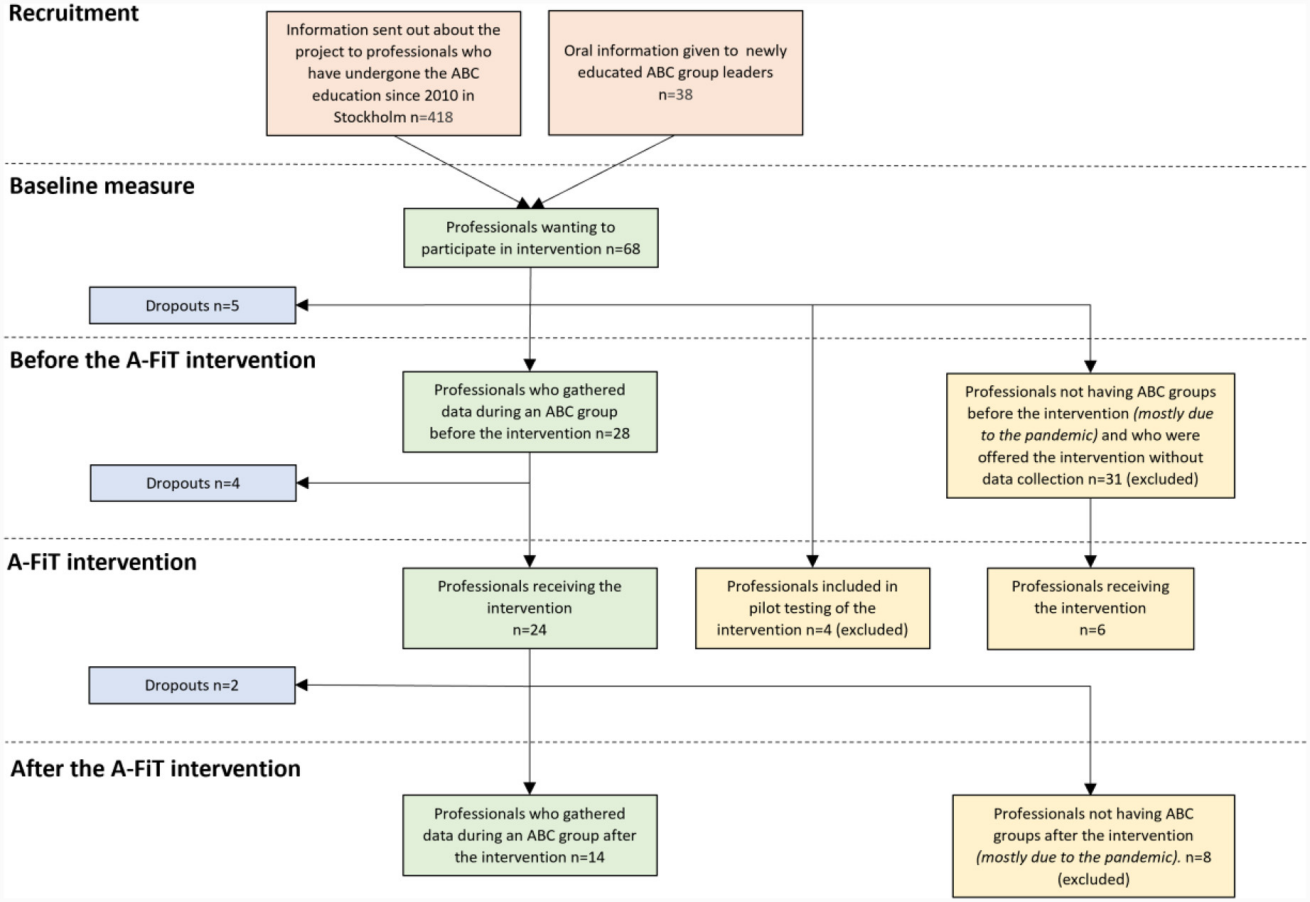
Flowchart of the Data Collection Process

Eligible study participants, referred to as *group-leaders,* were professionals trained to lead ABC groups. To be included, a group-leader had to answer a baseline questionnaire, hold an ABC group and be interviewed about adaptations made during the delivery of that group. The group-leader also had to participate in the A-FiT intervention and hold a new ABC group post-A-FiT with subsequent interviews (Figure 1). The primary recruitment occurred by reaching out through email to ABC alumni (*n* = 418) in Sweden. It is unknown how many of the ABC alumni were eligible to participate in the study, that is, were active as group-leaders during the recruitment period. Information about the study was also provided to new cohorts of ABC group-leaders during their ABC education during 2021–2022 (*n* = 38) by the first author. The recruitment period spanned May 2020 to June 2022, excluding a 7-month COVID-19 pandemic pause. A total of 103 group-leaders completed the baseline questionnaire (received after filling out an interest form or after listening to the oral information). Out of these, 68 group-leaders responded that they wanted to participate in A-FiT. A total of 14 group-leaders fulfilled all the inclusion criteria and were included in the study. The main reason for not fulfilling all inclusion criteria was not delivering ABC during the data collection period (May 2020–September 2023), mainly due to COVID-19 restrictions or other program commitments.

The mean age of the group-leaders was 49 years (SD: 9.6, range: 27–62), 86% (*n*: 12) were female and the experience of leading ABC groups varied between one and 11 years (mean: 5.7 years, SD: 2.8). Most group-leaders worked within social services (*n* = 8) or the school-environment (*n* = 5). Comparisons between these group-leaders and the larger baseline sample (*n*: 89) that didn't participate in A-FiT revealed no statistically significant differences in age, gender, working field, or ABC experience (*p*-value between .128 and .643).

### Data Collection

**Figure 2:**
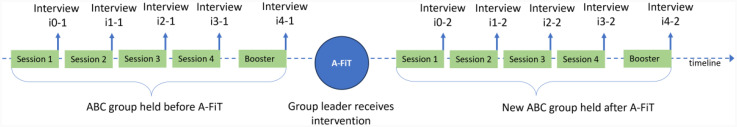
Timeline of the Data Collection

The adaptations made by each group-leader were tracked through brief, structured individual interviews, conducted within 1 week after the ABC-sessions they personally facilitated for a group of parents (Figure 2). Each group-leader facilitated four to five sessions per ABC group, resulting in four to five interviews both before and after A-FiT. The interview guide (Appendix 1) was based on an adaptation tracking sheet checklist (Rabin et al., 2018) and included questions about fidelity to and adaptations made of ABC (what, when, how, who, and why). In total, 127 interviews were conducted with 14 group-leaders (65 before and 62 after A-FiT), with an average of nine interviews per person.

All interviews were audiorecorded, conducted individually by the first author (JZ), as a video meeting or over the phone at a time convenient for the group-leader. The interviews lasted 4–55 min (mean about 13 min).

The planning, conducting, and reporting of this study were in accordance with the Helsinki Declaration (2013 revision). Ethical approval was obtained from the Swedish Ethical Review Authority (Ref No. 2019–06276). All participants were provided with written information about the study, including what participation entailed, how data was safeguarded, and how the participants could access the results. Voluntary participation was emphasized, including the option to withdraw at any time without explanation. All participants provided written informed consent, either electronically via the baseline questionnaire or by signing an attached document, depending on the recruitment method.

### Data Analysis

Using the single-phase mixed method approach, the data was first analyzed and coded using deductive content analysis (Krippendorff, 2019) and then counted and compared using a chi^2^-test. All interviews were transcribed verbatim and deductively analyzed, using a codebook (Appendix 2) developed from adaptation tracking sheet checklists (Rabin et al., 2018; Stirman et al., 2019). The codes used related to the questions and definitions of *what* (the nature of the adaptation (adaptation type)), *by whom* (who decided that the adaptation should be made and made the adaptation), and *why* (intentionality of the adaptation). To clarify the intentionality, participants explanations as to why adaptations were made were coded as *practical*, *higher-purpose*, or *nonintentional (i.e., unreflective). Practical* was applied for adaptations of how the ABC-sessions were conducted in relation to challenges of a practical nature to increase reach, participation, and so on. *Higher-purpose* was applied for adaptations aiming to increase or maintain the effectiveness of ABC. As some adaptations was reported to be made without an explicit reason, intention, or consideration of impact and conscious reasoning or purpose, these adaptations were coded as *unreflective*, in line with the definition of unreflective actions taken without critical thought or awareness (Mezirow, 1990). Additionally, codes exploring when (*“were the adaptation planned?”*) was used to clarify if the adaptations were planned prior to the ABC-session or made in reaction to unanticipated challenges during the session (coded as *planned* or *unplanned*). Finally, were the adaptations also coded based on fidelity consistency using the construct “*Relationship fidelity/core elements*.” This construct addressed whether the adaptation preserved or altered ABC´s core elements (each adaptation coded as *fidelity-consistent* or *fidelity-inconsistent*). To be able to code fidelity-consistency, ABC’s core elements were identified, as suggested by Stirman et al. (2019) by reviewing existing literature and evaluating available data on ABC and theories included in ABC (i.e., Bandura, 1977; Lindberg et al., 2013; Ulfsdotter, 2016) and input from treatment developers. Fidelity-consistent adaptations were defined as those that do not alter the core elements of ABC significantly enough to reduce adherence to the manual or reduce the ability to differentiate ABC from other EBIs (Stirman et al., 2015). Fidelity-inconsistent adaptations were defined as adaptations that reduce or preclude the delivery of ABC's core elements, decrease the ability to differentiate ABC from other treatments (Stirman et al., 2015), or alter the ability to deliver ABC as intended (e.g., missed sessions; Mui et al., 2023).

The codebook was developed and tested by JZ and EW who coded five interview transcripts each and compared and discussed the procedure and results. Discussions on codes and procedure were also held with all authors, resulting in refinements in code descriptions and additional examples of adaptations associated with a specific code.

### Coding and Analysis of dData

Each transcript was read through, and adaptations were identified and recorded in an Excel spreadsheet, one adaptation on each row. An adaptation was either identified as such by an interviewee stating it as an adaptation, or by an author reading the interviewees’ descriptions of actions taken and matching it to the criteria defining an adaptation (see above). The recorded adaptations were checked against the ABC manual to verify that they were adaptations and not accepted variations in ABC. Recording the adaptations consisted of transferring quotes from the interview data to the spreadsheet and coding each adaptation based on the codebook. To keep consistency in recording and coding, all interviews conducted with the same participant were analyzed one after the other by the same author. JZ conducted the majority of the coding, whereas EW coded 22% of the interviews. Continuous discussions were held between JZ and EW throughout the analysis to ensure consistency in coding and discuss and agree on how to code when uncertainties arose. Any refinements of the codes resulting from the discussions during the coding process were noted in the codebook. Once all adaptations had been coded and checked for accuracy in relation to the final version of the codebook, frequencies of occurrences of codes were calculated. To detect statistically significant changes in proportions of adaptations before and after A-FiT, a chi^2^-test was used, using IBM SPSS version 28.0.1.0. Exact *p*-values are presented, and in the text, *p* < .05 is referred to as significant.

Once descriptive statistics had been calculated and patterns in changes of adaptations before and after A-FiT had been found, the adaptations were sorted based on intentionality and analyzed along with planning and fidelity-consistency to better understand how A-FiT influenced the group-leaders’ adaptations. To gain a deeper understanding of what the adaptations in each combination of codes (planning, intentionality and fidelity consistency) consisted of and why these adaptations were made, the adaptations were re-read and compared. In the results, quotes from the group-leaders (translated into English) are used to illustrate the adaptations before and after A-FiT.

## Results

In total, the 14 group-leaders held 130 ABC-sessions, 65 before and 65 after A-FiT intervention. A total of 228 adaptations were made before (6–30 adaptations per group-leader, 0–11 adaptations per ABC-session) and 218 after A-Fit (7–33 per group-leader, 0–10 per ABC-session; Table 1).

**Table 1:** Adaptations Made Before and After the A-FiT Intervention

		**Before A-FiT** *N *(% within the column)	**% within row**	**After A-FiT** *N *(% within the column)	**% within row**	**Change between columns before versus after A-FiT**	**Chi^2^**	***df***	**Significance value** (*p*)
**The total number of adaptations made**		228 (100)		218 (100)		↓:10	.22	1	.636
**Adaptations made/group-leader**		6–30		7–33					
**Adaptations made/ABC-session**		0–11		0–10					
**Adaptation type**							25.5	11	.008
Substituting		51 (22)	56	40 (18)	44	↓4%			
Tailoring/tweaking/refining		45 (20)	52	42 (19)	48	↓1%			
Shortening/condensing/pacing/timing		39 (17)	50	40 (18)	50	↑1%			
Lengthening/extending		22 (10)	58	16 (7)	42	↓3%			
Integrating another framework into ABC		19 (8)	67	9 (4)	32	↓4%			
Removing/skipping elements		17 (7)	71	7 (3)	29	↓4%			
Loosening structure		17 (7)	38	28 (13)	62	↑6%			
Adding elements		12 (5)	48	13 (6)	52	↑1%			
Departing from ABC (drift)		5 (2)	19	21 (10)	81	↑8%			
Reordering		1 (0)	100	0 (0)	0	—			
Integrating ABC into another framework		0 (0)	0	1 (0)	100	—			
Repeating		0 (0)	0	1 (0)	100	—			
**Planning**							2.4	1	.125
Planned		142 (62)	54	120 (55)	46	↓7%			
Unplanned		86 (38)	47	98 (45)	53	↑7%			
**Intentionality**							6.4	2	.041
Practical		131 (57)	47	151 (69)	53	↑12%			
Higher-purpose		80 (35)	60	53 (25)	40	↓10%			
Unreflective		17 (7)	55	14 (6)	45	↓1%			
**Fidelity consistency**							2.1	1	.168
Fidelity-consistent		172 (75)	53	151 (69)	47	↓6%			
Fidelity-inconsistent		56 (25)	46	67 (30)	54	↑5%			

*Note.* A-FiT = the Adaptation and Fidelity Tool.

### Description of how many and what kind of Adaptations were Made Before and After A-FiT

The distribution of adaptations among the adaptation types after A-FiT was statistically significantly different from the distribution before A-FiT *X*^2^ (11, *N *= 446) = 25.5, *p *= .008. Some types of adaptations decreased in proportion after A-FiT, for example, the group-leaders reported less removal of elements, substituting, and integration of another framework into ABC. Examples of adaptations within these types were skipping role play (removal), digital format (substituting), and including elements from Komet, another parent education program (integrating another framework into ABC).

Some adaptation types also increased after A-FiT, such as loosening structure, and departing from ABC (drifts). These adaptation types consisted mostly of adaptations such as skipping the break to make up for time loss (loosening structure) and allowing children to participate during the sessions (departing from ABC).

The most common types of adaptations made of ABC before receiving A-FiT were substituting, tailoring, and shortening (Table 1). These types also remained the most common after receiving A-FiT, although the distribution among them was slightly altered. Within these types, examples of common adaptations made of ABC both before and after A-FiT were changing roleplay format (substituting), rephrasing content and questions (tailoring), and finishing an ABC-session early (shortening).

#### Intentionality of the Adaptations

More than half of the adaptations made, both before and after A-FiT, were due to practical reasons, making this intention the most common for making adaptations (Table 1). Yet, the proportions of the intentionality of adaptations changed significantly after A-FiT *X*^2^ (2, *N *= 446) = 6.4, *p *= .041, and the practical adaptations increased while adaptations made due to a higher purpose decreased. It was uncommon to make unreflective adaptations both before and after A-FiT and the proportion of these adaptations remained almost the same before versus after. It was noticeable that different types of unreflective adaptations were reported before versus after A-FiT, indicating the varying nature of these adaptations.

#### Planning of the Adaptations

The group-leaders made more planned than unplanned adaptations both before and after A-FiT, but the planned adaptations decreased and the unplanned increased after A-FiT (Table 1). However, there was no statistically significant change in the number of planned and unplanned adaptations after A-FiT.

#### Fidelity Consistency of the Adaptations

The majority of the adaptations were fidelity-consistent, both before and after A-FiT. The number of fidelity-consistent adaptations was higher before A-FiT than after (Table 1). Still, no statistically significant change was detected.

### How A-FiT Influenced the Group-Leaders’ Adaptations

To further understand the adaptations made both before and after A-FiT, the combination of intentionality and planning was investigated (Table 2). The analysis of the various combinations showed that it was mostly *planned, higher-purpose adaptations that decreased* after A-FiT, and that both *planned and unplanned practical adaptations increased* after A-FiT. Additionally, *fidelity consistency as a layer of intentionality and planning adaptations* was investigated to provide insight into whether the adaptations that increased or decreased were in line with the core elements or not.

**Table 2:** Adaptations Sorted Based on Intentionality and Combined with Planning and Fidelity Consistency

**Intentionality of adaptation**	**Planning-level**	**Before A-FiT** *N* (% within the column)	**After A-FiT** *N* (% within the column)	**Change in proportion between columns before versus after**	**Fidelity consistency**	**Before A-FiT*** N* (% within the column)	**After A-FiT** *N* (% within the column)	**Change in proportion between columns before versus after**
Practical	Planned	76 (33)	87 (40)	↑6%	Consistent	58 (25)	58 (27)	↑ 2%
					Inconsistent	18 (8)	29 (13)	↑5%
	Unplanned	55 (24)	63 (29)	↑5%	Consistent	33 (14)	40 (18)	↑4%
					Inconsistent	22 (10)	23 (11)	↑1%
Higher-purpose	Planned	60 (26)	28 (13)	↓13%	Consistent	57 (25)	24 (11)	↓14%
					Inconsistent	3 (1)	4 (2)	↑1%
	Unplanned	20 (9)	26 (12)	↑3%	Consistent	18 (8)	23 (11)	↑3%
					Inconsistent	2 (1)	3 (1)	
Unreflective	Planned	6 (3)	5 (2)	↓1%	Consistent	2 (1)	1 (0)	↓1%
					Inconsistent	4 (2)	4 (2)	
	Unplanned	11 (5)	9 (4)	↓1%	Consistent	4 (2)	5 (2)	
					Inconsistent	7 (3)	4 (2)	↓1%

*Note.* A-FiT = the Adaptation and Fidelity Tool.

#### Planned, Higher-Purpose Adaptations Decreased After A-FiT

The decrease in planned higher-purpose adaptations after A-FiT consisted of group-leaders reporting fewer additional examples or pictures (i.e., less tailoring), adjusting the role-playing format (i.e., less substituting), and integrating activities from other programs (i.e., less integration of another framework into ABC) after A-FiT. When analyzing the remaining higher-purpose adaptations after A-FiT, the group-leaders spoke about them removing adaptations they had previously added (Table 3, Quote 1).

**Table 3:** Quotes From the ABC Group-Leaders About Adaptations Made

**Quote number**	**Quote**	**Group-leader**	**Interview occasion**
Quote 1	“…I did not add from Komet (another parent education program). I might have mentioned the Komets flashlight and scale, but not as much as I have done before. Instead, I stuck more to the ABC manual//It felt more legitimate too. I think it is great because people have an ‘aha’ experience when you add it, but then I don’t follow the ABC manual … So, it felt more legitimate//I decided it beforehand. I probably understood that time would be short, but still, it was more like: I will stick more to the manual than I did before.”	No. 2	First interview after the A-FiT intervention
Quote 2	Group-leader: “The only thing I would say we added … is a balance scale from Komet. Because I find it, it helps to clarify what it is all about, finding a balance between, how should I say it, encouragement and love and care and concern//It is an image. Now I did not … or I have had it with me (when delivering ABC) before. Now I only drew it, so it is like one of those classic balance scales, like a seesaw//I just threw it in completely freely.” Interviewer: “So it was one of those things that just came up? You felt that you wanted to reinforce, in the moment, what you were talking about?” Group-leader: “Yes, exactly.”	No. 1	First interview after the A-FiT intervention
Quote 3	“…we have children who are … usually until they do not want to stay in the room, in the room with us, let's say up to one year old. So we probably had four children with us yesterday (during the ABC-session) … And that is to make it possible for the parents to participate (in ABC).”	No. 16	First interview after the A-FiT intervention
Quote 4	“It was during the program, the session. Not during the break, but during the time (the session was held). A lively boy who moved around the room like this, and when he approached, a dad started to play with him, and he (the dad), followed the boy onto a blanket on the floor and played with him a bit there. Then the boy crawled to another parent, and she picked him up and spoke to him soothingly like this, and he sat in her lap and looked at her and so on.”	No. 17	Second interview after the A-FiT intervention

*Note.* A-FiT = the Adaptation and Fidelity Tool.

As these planned higher-purpose adaptations decreased, the pattern slightly shifted from planned to unplanned higher-purpose adaptations. This change consisted of group-leaders making more unplanned higher-purpose adaptations of the adding elements type after A-FiT. These additions were described as adding information that the group-leaders had chosen to remove prior to the ABC-session but realized during the session that they still needed (Table 3, Quote 2).

#### Planned and Unplanned Practical Adaptations Increased After A-FiT

The increase in planned practical adaptations after A-FiT consisted mostly of group-leaders making adaptations aiming to accommodate parents’ needs. For example, group-leaders allowed children to participate during the ABC-sessions to ensure the parents’ participation, an adaptation not reported before A-FiT (Table 3, Quote 3).

The increase in unplanned practical adaptations reported after A-FiT consisted of adaptations mainly of the shortening/condensing and loosening structure types. Common examples of adaptations of these types, both before and after A-FiT, related to being short on time, forcing the group-leader to finish a session early (i.e., shortening/condensing), or skipping a break (i.e., loosening structure). However, after A-FiT the group-leaders to a greater extent reported that they found themselves short on time. Some of the reported unplanned practical adaptations after A-FiT also consisted of reactions to situations that occurred due to the inclusion of other planned practical adaptations. For example, the inclusion of children during a session (a planned practical adaptation), could, when the child was restless, force an unplanned practical adaptation whereby another parent or group-leader interacted and played with that child during the session to facilitate completion of the session (Table 3, Quote 4).

#### Fidelity Consistency as a Layer of Intentionality and Planning of the Adaptations Made

The combination of intentionality and planning together with fidelity consistency provided insight into why the results indicate that the group-leaders made more fidelity-inconsistent and less fidelity-consistent adaptations after A-FiT. Almost all of the planned, higher-purpose adaptations that deceased after A-FiT were fidelity-consistent. This decrease in planned, higher-purpose adaptations contributed to the decrease in fidelity consistency adaptations. Furthermore, many of the practical, planned adaptations that increased after adaptations, such as allowing children to participate, were primarily categorized as departing from the intervention making these adaptations’ fidelity-inconsistent. The increase in practical, planned adaptations also contributed to an increase in fidelity-inconsistent adaptations.

## Discussion

The group-leaders made about the same number of adaptations before and after A-FiT, indicating that A-FiT did not impact the total *number* of adaptations. However, participation in A-FT lead to statistically significant changes in adaptation *types*, and to a lower level of *planned higher-purpose* adaptations. Also, both *planned* and *unplanned* adaptations made for a *practical* reason increased after A-FiT. Finally, no statistically significant change was observed in the *fidelity consistency* of the adaptations, although the proportion changed slightly as many of the higher-purpose adaptations that decreased after A-FiT were fidelity-consistent.

The changes in the *type,* but also *intentionality,* of adaptations can indicate that the group-leaders were more aware of fidelity and adaptations after participating in A-FiT. A prior study showed that A-FiT increased professionals’ knowledge of fidelity and adaptations (Hasson et al., 2023). In this study, that knowledge is reflected in a decrease in planned higher-purpose adaptations and the ambition to adhere to the ABC manual, combined with an increase in unplanned higher-purpose adaptations. Although these findings might seem ambiguous, the decrease in planned higher-purpose adaptations may indicate that many adaptations introduced before A-FiT had become routine, without a reassessment of their necessity. Thus, after A-FiT, some of these adaptations were deemed unnecessary upon further reflection and removed, leading to fewer planned higher-purpose adaptations. However, some of these removed adaptations were reintroduced in the moment if they were considered essential to maintain effectiveness, which may explain the increase in unplanned higher-purpose adaptations after A-FiT. These observations suggest an increased awareness among professionals of the adaptations made after A-FiT and align with the idea that EBIs need to be adapted and evolve to maintain effectiveness during the sustainment phase (Chambers et al., 2013; Glasgow et al., 1999). Additionally, the finding that adaptations implemented with a specific purpose were not always revisited highlights the importance of continuously reflecting on decisions about both current and previously made adaptations. To refine A-FiT in the future, questions that prompt reflection on existing adaptations and their relevance could be included.

The professionals’ increased awareness of fidelity and adaptations after A-FiT might also be indicated by the rise in planned practical adaptations, reflecting an understanding of how adaptation and fidelity relate to outcomes and values, in line with the Value Equation (von Thiele Schwarz et al., 2019).

Based on the Value Equation, the A-FiT workshops encouraged the group-leaders to consider the broader impact of adaptations and fidelity on multilevel values. Such consideration could be seen through the increase in planned practical adaptations found after A-FiT, as many of these adaptations aimed for new outcomes and values (such as increasing reach by letting children participate during a session) indicating a shift in the outcomes and values sought by the group-leaders.

Many of the *planned practical adaptations* that increased after A-FiT tended to be *fidelity-inconsistent*. Although fidelity-inconsistent adaptations have been found to reduce inequalities in access and use of care for a specific target group (Aschbrenner et al., 2020, 2021), they are often considered unwanted. This unwantedness is based on the assumption that high fidelity is related to increasing quality and is often considered a primary outcome in implementation research (Proctor et al., 2011). Yet, a fidelity-inconsistent adaptation may not automatically make the adaptation inappropriate (von Thiele Schwarz et al., 2019). For example, enabling parents without childcare to attend may help reach more parents who might benefit by participating in ABC. This illustrates that the evaluation of whether an adaptation is appropriate or not may only be determined based on a careful consideration of the tradeoffs between different outcomes (e.g., clinical outcomes or reach). This corresponds with strategies focused on maximizing public health outcomes, which recognize that an EBI with a smaller effect size can still lead to significant public health benefits if it can reach a broader patient population and is sustained over time (Rutledge & Loh, 2004).

That the *planned* and *unplanned practical adaptations* increased after A-FiT points to a possible “ripple effect” (Kirk et al., 2020). The ripple effect is when a single adaptation affects multiple outcomes (or values), which can be both intended and unintended. For example, when the group-leaders made *planned practical adaptations,* unintended situations or outcomes sometimes led to a new situation, “forcing” the group-leaders to make *unplanned practical adaptations.* The findings confirm that making adaptations may have unforeseen consequences which need to be considered when adaptations are made.

A-FiT did not impact the total number of adaptations. However, combinations of intentionality, planning, and fidelity consistency of adaptations give a better understanding of the complexity of adaptations rather than counting numbers. For example, the total number of adaptations can confound the calculation due to the so-called “solar eclipse situation.” We found that one single adaptation can eliminate other adaptations that might have been made. An example is when a group-leader reported skipping the booster session; this one adaptation removed a whole session and thereby eliminated the possibility of making adaptations during that session and consequently, fewer adaptations were reported. Thus, “The solar eclipse situation” indicates that it is insufficient to only look at the numbers of adaptations, instead it is important to understand each adaptation's impact and complexity.

The method of coding planning and intentionality separately in this study makes a meaningful contribution to earlier research. FRAME suggests an inextricable link between process (planning) and drive (reactive/proactive) which indirectly states that reactive adaptations cannot be systematic and planned (Kirk et al., 2020). Reactive adaptations occur during the delivery of a program, often due to unanticipated obstacles (Moore et al., 2013). This study shows, however, that many adaptations, while unplanned, had a clear purpose. This suggests that categorizing adaptations as proactive/reactive is insufficient during the sustainment phase, particularly for professionals who have been trained to consider their adaptations in relation to their intended or anticipated impact. Thus, planning does not necessarily indicate the process of the adaptations; instead, it can indicate whether many unanticipated situations occur when working with a specific EBI, highlighting the need to prepare for various scenarios when using EBIs during the sustainment phase.

## Methodological Discussion

There are some important limitations to note. Firstly, the interviews may have influenced the group-leader's way of thinking about and managing adaptations, potentially impacting internal validity. Secondly, participants may have hesitated to volunteer information about adaptations, particularly before participating in A-FiT, as fidelity to EBIs is an integral part of professional training. Also, participants with long experience of ABC might not remember fully if their way of delivering ABC deviated from the manual. Both these circumstances may have led to an underestimation of adaptations before A-FiT. However, using prompts during interviews and having the same person conducting all interviews enabled the formation of a trusting relationship between the participant and the author, which may have circumvented this.

The coding of adaptations might have impacted the frequency of adaptations reported in the results. To ensure rigor in coding, a codebook was developed and used, with continuous discussion among coders. For codes (like intentionality) developed for this study and not previously tested, extra attention was given when coding to safeguard trustworthiness. Still, as with any qualitative research, the coding of the participants responses was based on the authors’ interpretation of their answers and the authors have aimed for transparency in the interpretation of the findings.

To safeguard the integrity of the interview guide and the structured interview process the research team continuously discussed how the interviews were conducted during the data collection period. The authors also have expertise in qualitative and quantitative methods and implementation science which strengthens the study's creditability.

This study was performed during the COVID-19 pandemic, which caused challenges with recruitment and data collection, as many group-leaders were forced to focus on other work tasks than delivering ABC. However, the within-person design used in this study is suitable when having small samples and following participants over an extended period. The richness of the information gained using the tracking approach also makes it possible to study adaptations and decisions related to adaptations thoroughly.

Furthermore, the decision to employ the tracking sheet as an interview guide was influenced by pilot-testing it as a questionnaire, using cognitive interviews, both of which align with its suggested use. Pilot results revealed that the questionnaire was cumbersome, potentially increasing the risk of underreported adaptations or participation dropouts (Miller-Day et al., 2013). By using an interview approach, the study captured a broad range of adaptations, facilitating participant reflection and minimizing omissions. While this approach might seem extensive, a pragmatic focus on major adaptations to reduce participant burden was deemed insufficient as distinguishing major and minor adaptations was not straightforward in this study. Until more is known about the impact of different adaptations, our comprehensive method provides important groundwork for identifying key adaptations.

This study used self-reported data, which can lead to underreporting compared to observational methods (Miller-Day et al., 2013). However, the use of prompts helped enhance the quality of the data by providing valuable reflective depth. Future research could incorporate observational methods to verify adaptations, but these alone may lack the reflective insights essential for understanding the reasoning and planning behind adaptations.

Potential selection bias may affect the results if only group-leaders interested in fidelity and adaptation questions chose to participate in the study. The results may thus represent professionals who are somewhat more aware of adaptations than professionals in general. Yet, the participants were diverse in terms of occupation, age, and experience, and did not differ from the professionals who did not want to participate in the study, which strengthens the study's generalizability. All participants worked with the same program, had been trained, and were supported by the same purveyor, which was necessary for the study to carefully track adaptations.

## Conclusion

This study investigates adaptations made by group-leaders delivering an evidence-based parental program in the sustainment phase of implementation, both before and after receiving A-FiT, a decision support intervention. The findings revealed that A-FiT did not significantly alter the overall number of adaptations made by the group-leaders, but it did lead to significant changes in the type and intentionality of the adaptations made. The results suggest that A-FiT contributes to increased awareness about fidelity and adaptation among the group-leaders, making it a valuable tool for supporting professionals when handling fidelity and adaptation. Additionally, the research highlights the complexities of adaptations in the implementation of EBIs, emphasizing a need for a nuanced understanding of the types, intentions, fidelity, and consequences.

## Supplemental Material

sj-docx-1-irp-10.1177_26334895251334552 - Supplemental material for Evaluating professionals’ adaptations before and after a decision support intervention “the Adaptation and Fidelity Tool” (A-FiT)—A longitudinal within-person intervention design Supplemental material, sj-docx-1-irp-10.1177_26334895251334552 for Evaluating professionals’ adaptations before and after a decision support intervention “the Adaptation and Fidelity Tool” (A-FiT)—A longitudinal within-person intervention design by Johanna Zetterlund, Henna Hasson, Ulrica von Thiele Schwarz, Margit Neher and Emmie Wahlström in Implementation Research and Practice

sj-docx-2-irp-10.1177_26334895251334552 - Supplemental material for Evaluating professionals’ adaptations before and after a decision support intervention “the Adaptation and Fidelity Tool” (A-FiT)—A longitudinal within-person intervention design Supplemental material, sj-docx-2-irp-10.1177_26334895251334552 for Evaluating professionals’ adaptations before and after a decision support intervention “the Adaptation and Fidelity Tool” (A-FiT)—A longitudinal within-person intervention design by Johanna Zetterlund, Henna Hasson, Ulrica von Thiele Schwarz, Margit Neher and Emmie Wahlström in Implementation Research and Practice

sj-pdf-3-irp-10.1177_26334895251334552 - Supplemental material for Evaluating professionals’ adaptations before and after a decision support intervention “the Adaptation and Fidelity Tool” (A-FiT)—A longitudinal within-person intervention design Supplemental material, sj-pdf-3-irp-10.1177_26334895251334552 for Evaluating professionals’ adaptations before and after a decision support intervention “the Adaptation and Fidelity Tool” (A-FiT)—A longitudinal within-person intervention design by Johanna Zetterlund, Henna Hasson, Ulrica von Thiele Schwarz, Margit Neher and Emmie Wahlström in Implementation Research and Practice

sj-pdf-4-irp-10.1177_26334895251334552 - Supplemental material for Evaluating professionals’ adaptations before and after a decision support intervention “the Adaptation and Fidelity Tool” (A-FiT)—A longitudinal within-person intervention design Supplemental material, sj-pdf-4-irp-10.1177_26334895251334552 for Evaluating professionals’ adaptations before and after a decision support intervention “the Adaptation and Fidelity Tool” (A-FiT)—A longitudinal within-person intervention design by Johanna Zetterlund, Henna Hasson, Ulrica von Thiele Schwarz, Margit Neher and Emmie Wahlström in Implementation Research and Practice
