# Supplementary material for: Evaluating professionals’ adaptations before and after a decision support intervention “the Adaptation and Fidelity Tool” (A-FiT)—A longitudinal within-person intervention design
Source: Implement Res Pract. 2025 Apr 13;6:26334895251334552. doi: 10.1177/26334895251334552 (PMC12033404; doi:10.1177/26334895251334552)
Supplement: sj-docx-1-irp-10.1177_26334895251334552 - Supplemental material for Evaluating professionals’ adaptations before and after a decision support intervention “the Adaptation and Fidelity Tool” (A-FiT)—A longitudinal within-person intervention design [file sj-docx-1-irp-10.1177_26334895251334552.docx]

**Appendix 1.**

Interview guide

1. How easy or difficult was it to follow the ABC manual during this ABC session?
2. What made it easy/difficult?
3. Did you do anything special to follow the ABC manual?
4. Did you make any adaptations during/off this session?

If yes:

a). What did you do?

b). What was the intention of the adaptation and what was it expected to lead to?

c). Who, if not you, made decisions about the adaptation?

*E.g.: You/colleague/manager/authority/participant*

d). When was the adaptation made?

*E.g.: Before/during/after the meeting*

I will now give you some examples of adaptations that could have been made, which one might not think of spontaneously. Did you do any of the following? *(If yes, ask question 4a-d for each adaptation).*

- Removed or added parts/elements.
- Changed length and/or intensity of the program.
- Replaced or combined the program/parts of the program with other methods/other information.
- Gave the parts in a different order.
- Repeated the program or selected parts of the program.
- Stepped away from the manual.
- Gave the program in another context (e.g., another type of business).
- Gave the program in a different format (e.g., individually/fewer participants or via the Internet).
- Offered the program in a location other than originally intended.
- More/fewer than two group-leaders provided the program.
- The program was provided by group-leaders with prior knowledge.
- The program was used for a different target group.
- Can you think of any other/more adaptations?

**Additional questions asked at the fourth ABC session:**

1. Will you have a booster session?

*If yes: book an interview and ask the same questions as above on that occasion.*

*If no*: a). How come?

b). What was the intention of not holding the booster session?

c). Who, if not you, made decisions about the adaptation?

*E.g.: You/colleague/manager/authority/participant*

e)*.* Did you do something else instead of the booster session?

*E.g.: Replaced it with emails or offers of individual follow-up?*

f)*.* Did you do anything special to be able to adhere to ABC when you e.g. replaced the booster session?

g). What was the intention of the adaptation and what was it expected to lead to?

h). Who, if not you, made decisions about the adaptation?

*E.g.: You/colleague/manager/authority/participant*
