# Supplementary material for: Evaluating professionals’ adaptations before and after a decision support intervention “the Adaptation and Fidelity Tool” (A-FiT)—A longitudinal within-person intervention design
Source: Implement Res Pract. 2025 Apr 13;6:26334895251334552. doi: 10.1177/26334895251334552 (PMC12033404; doi:10.1177/26334895251334552)
Supplement: sj-docx-2-irp-10.1177_26334895251334552 - Supplemental material for Evaluating professionals’ adaptations before and after a decision support intervention “the Adaptation and Fidelity Tool” (A-FiT)—A longitudinal within-person intervention design [file sj-docx-2-irp-10.1177_26334895251334552.docx]

Appendix 2.

Codebook

| **Construct according to Rabin et al. (2018) or FRAME (Stirman et al., 2019)** | **Titel on each column in excel file** | **Coding categories according to Rabin et al/FRAME or other** | **Clarification of coding categories according to Rabin or FRAME** | **Clarification of coding category in relation to ABC** |
| --- | --- | --- | --- | --- |
| **WHAT is modified?** | **3a. What is modified**  (free text) | *Free text* | Quotations from the text (and extra clarification if needed) of what was modified |  |
|  | **3b. What is modified**  (summarized in a few words) | *Free text* | Modification summarized in a few words |  |
|  | **3c. What is modified**  (content, context, training, and evaluation) | Content | Modifications made to content itself, or that impact how aspects of the treatment are delivered |  |
|  |  | Context | Modifications made to the way the overall treatment is delivered. |  |
|  |  | Training and evaluation | Modifications made to the way that staff are trained in or how the intervention is evaluated |  |
| **WHAT is the nature**  **of the content**  **modification?** | **5. Adaptation type** | Tailoring/tweaking/refining |  |  |
|  |  | Adding elements |  |  |
|  |  | Removing/skipping elements |  |  |
|  |  | Shortening/condensing (pacing/timing) |  |  |
|  |  | Lengthening/extending (pacing/timing) |  |  |
|  |  | Substituting |  |  |
|  |  | Reordering of intervention modules or segments |  |  |
|  |  | Integrating into another framework (e.g., selecting elements) |  |  |
|  |  | Integrating another treatment into EBP (not using the whole protocol and integrating other techniques into a general EBP approach) |  |  |
|  |  | Repeating elements or modules |  |  |
|  |  | Loosening structure |  |  |
|  |  | Departing from the intervention (“drift”) |  |  |
| **WHY: What was the purpose of the adaptation? (part 1)** | **7a. What was the purpose of the adaptation**  (free text) | *Free text* | Quotations from the text (and extra clarification if needed) of what was the purpose of the adaptation |  |
|  | **7b. What was the purpose of the adaptation**  *(condensed into a few words)* | *Free text* | The purpose summarized in a few words |  |
|  | **7c. What was the purpose of the adaptation** (reach, effectiveness, adoption, implementation) | Increase reach/participation/access |  |  |
|  |  | Increase effectiveness (or efficacy) |  |  |
|  |  | Increase adoption by more clinics/settings or make intervention more  aligned with organizational goals |  |  |
|  |  | Increase implementation/ability of staff to deliver intervention  successfully |  |  |
|  |  | Other |  | When a parent who participate in ABC “forces” an adaptation to be made with another purpose |
|  | **7d. What was the purpose of the adaptation (**Intentionality) | Intentional for a practical purpose |  | Intentional adaptations made to adapt how the ABC-sessions were conducted in relation to challenges of a practical nature. |
|  |  | Intentional for a higher purpose |  | Adaptations with the intention of increasing effectiveness of ABC |
|  |  | Unintentional/unreflective |  | Adaptations made without an explicit reason, intention, or consideration of impact and conscious reasoning or purpose, in line with the definition of unreflective actions taken without critical thought or awareness (Mezirow, 1990). |
| **BY WHOM was**  **adaptation made?** | **8a. BY WHOM was**  **adaptation made?**  (who took the initiative) | Individual practitioner/facilitator |  | Group leader |
|  |  | Colleague |  | Ex. Co-ABC group leader |
|  |  | Team |  | Ex. Colleagues not holding ABC with the group leader |
|  |  | Non-program staff |  | Ex. Manager |
|  |  | Administration |  | Ex. ABC coordinator |
|  |  | Program developer/purveyor |  | Ex. ABC developer/PLUS |
|  |  | Researchers |  |  |
|  |  | Coalition of stakeholders |  |  |
|  |  | Unknown/unspecified |  |  |
|  |  | ADDED: parent |  | Parent who participates in ABC |
|  | **8b. By WHOM was adaptation made?**  (who made the adaptation) | Individual practitioner/facilitator |  | The interviewed group leader |
|  |  | Colleague |  | Co-ABC group leader |
|  |  | Team |  | Ex. Colleagues not holding ABC with the group leader |
|  |  | Non-program staff |  | Ex. Manager |
|  |  | Administration |  | Ex. ABC coordinator |
|  |  | Program developer/purveyor |  | Ex. ABC developer/PLUS |
|  |  | coalition of stakeholders |  |  |
|  |  | Unknown/unspecified |  |  |
|  |  | Added: parent |  | Parent who participated in ABC |
| **Was the adaptation planned?**  Added and modified from FRAME (proactive/planned and reactive/unplanned)  (Stirman et al., 2019) | **9. Was the adaptation planned?**  (planned/unplanned) | Planned | “Adaptations are typically made proactively through a planning process that identifies ways to maximize fit and implementation success while minimizing disruption of the intervention” | The adaptation was planned before the ABC session began |
|  |  | Unplanned | “Occur during the course of program implementation, often due to unanticipated obstacles” | Before the ABC session started the adaptation was not planned to be made |
| **Relationship fidelity/core/elements?**  Added and modified from FRAME (Stirman et al., 2019) | **10. Relationship fidelity/core elements** | **Consistent** | Fidelity consistent adaptations were defined as those that do not alter the core elements of the EBI significantly enough to reduce adherence to the manual or reduce the ability to differentiate ABC from other EBIs (Stirman et al., 2015). |  |
|  |  | Inconsistent | Fidelity inconsistent adaptations were defined as adaptations that reduce or preclude the delivery of the EBI's core elements, decrease the ability to differentiate ABC from other treatments (Stirman et al., 2015), or alter the ability to deliver ABC as intended (e.g. missed sessions) (Mui et al., 2023). |  |

Mezirow J. (1990). Fostering critical reflection in adulthood: A guide to transformative and emancipatory learning. Jossey-Bass.

Mui, H.Z, Brown-Johnson, C.G, Saliba-Gustafsson, Lessios, A.S., Verano, M., Siden, R., Holdsworth, LM. (2023). Analysis of FRAME data (A-FRAME): An analytic approach to assess the impact of adaptations on health services interventions and evaluations. *Learning Health System, 8, 1*. https://doi.org/[10.1002/lrh2.10364](https://doi.org/10.1002/lrh2.10364)

Rabin, B. A., McCreight, M., Battaglia, C., Ayele, R., Burke, R. E., Hess, P. L., Frank, J. W., & Glasgow, R. E. (2018). Systematic, Multimethod Assessment of Adaptations Across Four Diverse Health Systems Interventions. *Frontiers in public health*, *6,* 102. https://doi.org/10.3389/fpubh.2018.00102

Stirman, S. W., Baumann, A. A., & Miller, C. J. (2019). The FRAME: An expanded framework for reporting adaptations and modifications to evidence-based interventions. *Implementation Science,* *14*, 1–10. <https://doi.org/10.1186/s13012-019-0898-y>

Stirman, S., Gutner, C., Crits-Christoph, P., Edmunds, J., Evans, A., & Beidas, R. (2015). Relationships between Clinician-Level Attributes and Fidelity-Consistent and Fidelity-Inconsistent Modifications to an Evidence-Based Psychotherapy. *Implementation Science*, *10*, 115. <https://doi.org/10.1186/s13012-015-0308-z>
